# A first insight into the genomic diversity of *Leptospira* strains isolated from patients in Cuba

**DOI:** 10.1371/journal.pone.0229673

**Published:** 2020-02-27

**Authors:** Angel A. Noda, Linda Grillová, Jean-Francois Mariet, Nadiezda B. Paiffer, Yanet C. Ruiz, Islay Rodríguez, Eduardo Echevarría, Ana Margarita Obregón, Reto Lienhard, Mathieu Picardeau

**Affiliations:** 1 Department of Mycology-Bacteriology, Institute of Tropical Medicine Pedro Kourí, Havana, Cuba; 2 Biology of Spirochetes Unit, Institut Pasteur, Paris, France; 3 Finlay Vaccine Institute, Havana, Cuba; 4 ADMED Microbiologie, La Chaux-de-Fonds, Switzerland; University of Kentucky College of Medicine, UNITED STATES

## Abstract

Leptospirosis is a neglected disease causing severe infections in humans and animals. Due in part to misdiagnosis, this infectious disease results in nearly 60,000 deaths per year around the globe. This study represents the first effort to describe the diversity of pathogenic *Leptospira* in Cuba based on whole-genome sequencing. We have collected nineteen whole-blood samples from patients that were diagnosed as having leptospirosis between 2008 and 2012 in Cuba. In addition, we have enhanced our sample set by three historical strains that were used for the development of a human vaccine in 1990s. The *Leptospira* strains were grown and serotyped by the microscopic agglutination test, and the draft genomes were generated by NGS (Illumina). Subsequently, the core genomes were analyzed and compared to the genetic data available from other Caribbean islands and countries in Central America. Core genome Multi-locus Sequence Typing (cgMLST) revealed four different core genome clonal groups (cgCGs), with the highest number of samples belonging to *L*. *interrogans*, followed by *L*. *borgpetersenii* and *L*. *kirschneri*. All cgCGs that were found in Cuba have been also identified from multiple origins across the globe, except in neighbor countries and Central America. Serotyping divided the samples into the serogroups Canicola, Ballum and Pomona. The most frequent cgCGs, cgCG28, associated with serogroup Canicola, and cgCG15, associated with serogroup Ballum, have also been identified from samples isolated from dogs, rodents, and pigs; suggesting that these hosts represent the major source of human infection in Cuba. The vaccine strains did not significantly differ from the recent patient isolates. However, the increasing prevalence of samples belonging to the serogroup Ballum combined with the fact that the available vaccine in Cuba represents inactivated *Leptospira* belonging to serogroups other than Ballum, should be a valuable information for the National and Regional Leptospirosis Control Programs.

## Introduction

Leptospirosis is a widespread, and potentially fatal, zoonosis that is endemic in many tropical regions and usually causes large epidemics after rainfall and flooding. Leptospirosis causes more than one million severe cases and 60,000 deaths every year worldwide. Infection usually results from exposure to an environment contaminated with the urine of infected reservoir host animals that carry the pathogen in their renal tubules [1].

Characterization of leptospiral isolates requires identification both at the species and serovar level. Sixty-four species have been identified by whole genome sequencing [2] while more than 300 serovars are defined by agglutination after cross-absorption of rabbit antisera with heterologous antigens [3]. Serovars that are antigenically related have traditionally been grouped into serogroups for convenience. Serogroups have no taxonomic standing, but they have proved useful for initial serological diagnosis and for epidemiological understanding at the regional or population level [3].

The first case of leptospirosis in Cuba was reported back in 1945 [4]. Cuba’s national leptospirosis prevention and control program was established in 1964 based on research carried out during a large outbreak among sugarcane workers in rural areas. The program has a large inter-sectoral component including the Ministry of Public Health, the Veterinary Medicine Institute, and other social and political organizations. The objective of the program is to control leptospirosis in both humans and animals. After a high number of human cases (2,828) reported in 1994, an emergency plan was developed and the Cuban vaccine was produced and evaluated in a preclinical phase. After the finalization of the clinical trial on volunteers, the vaccine was distributed countrywide [5].

This first vaccine in Cuba, vax-SPIRAL^®^, registered in 1998, was included within the National Leptospirosis Prevention and Control Program, and demonstrated its contribution to a morbi-lethality reduction of 82.1% [4]. The vaccine is based on bacterins which are inactivated *Leptospira* that present a series of drawbacks, such as serogroup-specific protection and short-term immunity.

The absence of available molecular typing data and overall incomplete picture of genomic diversity of *Leptospira* strains in Cuba has motivated us to type strains isolated from patients diagnosed as having leptospirosis in Cuba (2008–2012) using the recently proposed core genome multi-locus sequence typing (cgMLST) [6]. This report represents the first effort to describe the genetic diversity of pathogenic *Leptospira* in Cuba and the comparison of the MLST data of strains from other Caribbean islands and countries in Central America.

## Materials and methods

### Collection and in vitro cultivation of *Leptospira* strains

Nineteen whole-blood samples were collected from patients that were diagnosed as having leptospirosis during 2008–2012 at the Institute of Tropical Medicine Pedro Kourí (IPK, Havana, Cuba) (S1 Table). All patients had been diagnosed based on clinical symptoms, epidemiological picture, and serological tests. *Leptospira* was cultured by inoculating one and two blood drops into 5mL of commercial EMJH liquid medium (BD Difco^™^ Leptospira Medium Base EMJH, Difco^™^ Leptospira Enrichment EMJH). Blood cultures were incubated in the dark at 30°C for several weeks and tubes were checked weekly by dark-field microscopy.

### Serotyping

Strains isolated from patients (n = 19) as well as historical strains used for the vaccine development (n = 3) were subjected to the microscopic agglutination test (MAT) at the National Laboratory for Leptospirosis at the IPK and Finlay Vaccine Institute (IFV, Havana, Cuba). MAT was performed using polyclonal rabbit immune sera for the 12 most prevalent serogroups in Cuba and monoclonal antibody panels (Royal Tropical Institute, Holland) for the serovar identification, as established by the National and International Guidelines for the Diagnosis of Leptospirosis [7].

### Next-generation sequencing (NGS)

DNA was extracted from culture using the PureLink® Genomic DNA Mini kit (Invitrogen, Dublin, Ireland) and Wizard® Genomic DNA Purification Kit (Promega, Southampton, UK) according to manufacturer instructions.

NGS was performed using Nextera XT DNA Library Preparation kit and the NextSeq 500 sequencing systems (Illumina, San Diego, CA, USA) at the Mutualized Platform for Microbiology (P2M) at Institut Pasteur. The data were analyzed using CLC Genomics Workbench 9 software (Qiagen, Hilden, Germany) (S2 Table). Sequences, along with the sample metadata, were submitted to the publicly available BIGSdb hosted at Institut Pasteur MLST and whole genome MLST databases (Bacterial Isolate Genome Sequence database, https://bigsdb.pasteur.fr/leptospira/).

### Core genome Multi-Locus Sequence Typing (cgMLST)

The core genomes of the examined isolates were determined using BIGSdb as described previously [6]. Briefly, 545 core genes were extracted, concatenated, and analyzed in order to determine the core genome Sequencing Type (cgST) and core genome Multi-Locus Sequence Typing Clonal Groups (cgMLST CGs).

### Phylogenetic analyses

Maximum likelihood phylogeny was done using MEGA (v6.0) [8] with the Tamura-Nei model and 100 bootstrap replicates. The visualization of the phylogenetic trees was completed using iTOL (v4) [9]. To compare sequencing data obtained from Cuba with all available sequencing data obtained from the Caribbean islands and Central America, we extracted the six genes (*glm*U, *pnt*A, *suc*A, *tpi*A, *pfk*B, *mre*A, *cai*B) that are part of the MLST 1 scheme [10] from our raw data and enhanced our sample set by additional sequences stored at publically available Institut Pasteur MLST and pubMLST databases (S3 Table). This samples set included isolates from Barbados (n = 5), Guadalupe (n = 5), Trinidad and Tobago (n = 1), Costa Rica (n = 3), Panama (n = 14), Martinique (n = 3), Jamaica (n = 1), Nicaragua (n = 1), and Puerto Rico (n = 2).

### Ethics statement

The study was approved by the Research Ethics Committee of the IPK (CEI-IPK 87–17) and it was conducted in compliance with the Declaration of Helsinki. All participants provided written informed consent.

## Results

We sequenced the *Leptospira* genomes isolated from nineteen clinical samples of patients having leptospirosis during 2008–2012 in Cuba. Most of the patients (n = 16) came from the Holguín province, two patients came from Las Tunas and one from Havana (S1 Table). To study the dynamics of *Leptospira* infection in Cuba, we additionally sequenced three historical strains of *Leptospira*, which were used for the original vaccine development in the 1990s. NGS data displayed 104–462 contigs per genome with mean lengths ranging from 8.671 kbp to 37.554 kbp, and with the N50 length of contigs ranging from 13.434 kbp to 67.113 kbp (S2 Table).

**Fig 1:**
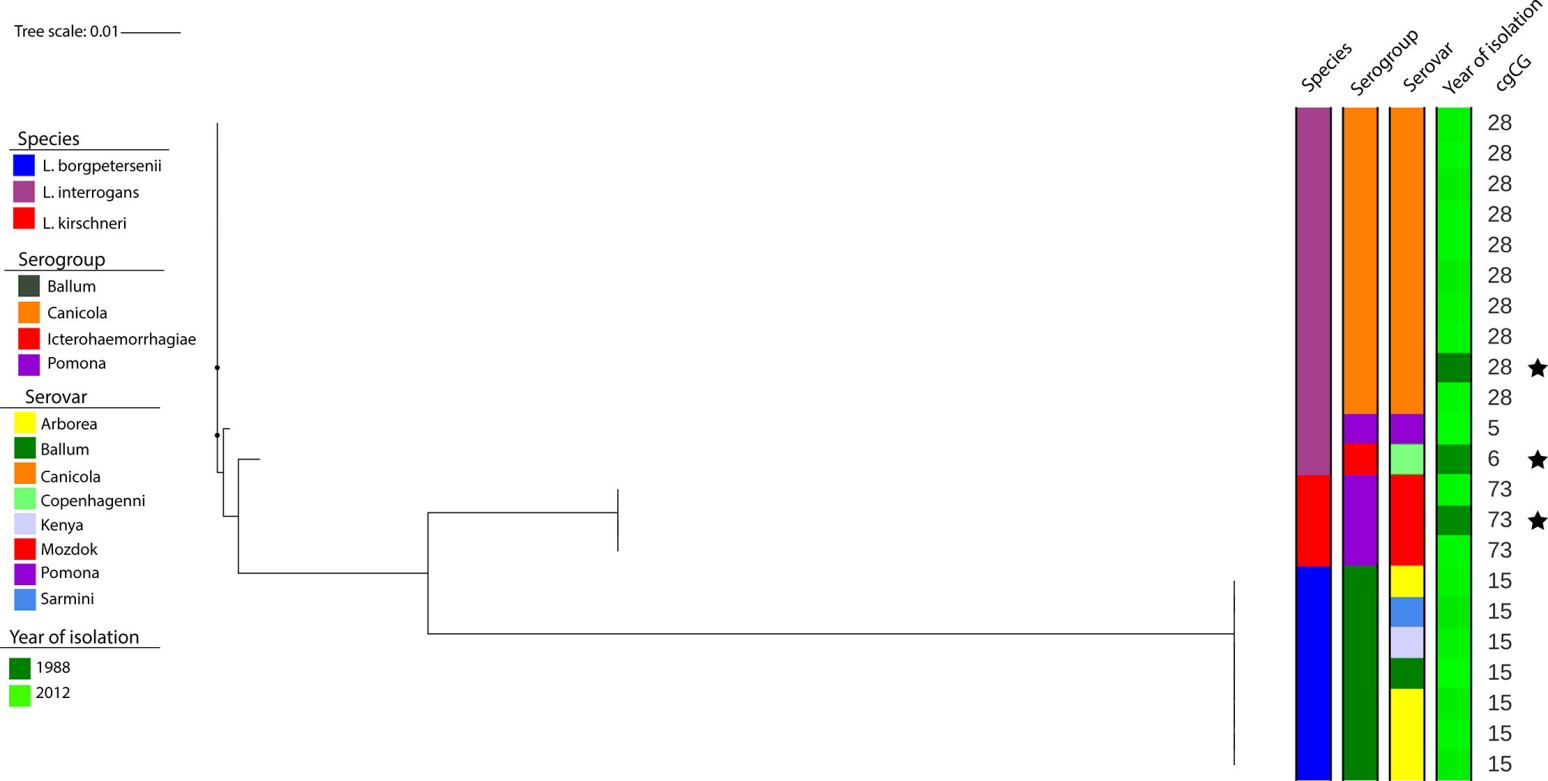
Phylogeny of samples isolated from 19 patients with leptospirosis hospitalized in Cuba during 2008–2012 and historical strains used for the vaccine development in the 1990s. Maximum likelihood bootstrapping was used to generate the phylogenetic tree based on 116,704 variable positions found in the 545 core genes. Metadata such as species, serogroup, serovar, isolation year, and clonal group are given next to the branches. The historical strains used for the vaccine development are marked by stars.

Analyses of core genomes divided the sample sets isolated from the current human population in Cuba into three species and four clonal groups (CGs) (Fig 1, S1 Table). The most predominant clonal group was CG28 (47.4%, 9/19), followed by CG15 (36.8%, 7/19), CG73 (10.5%, 2/19) and CG5 (5.3%, 1/19). We have identified *L*. *interrogans* as the most predominant species (52.6%, 10/19), followed by *L*. *borgpetersenii* (36.8%, 7/19) and *L*. *kirschneri* (10.6%, 2/19). Serotyping divided the examined samples into the following serogroups: i.) Canicola (47.4%, 9/19); ii.) Ballum (36.8%, 7/19) and iii.) Pomona (15.8%, 3/19) (Fig 1). The historical strains used for the vaccine development belonged to the CG28 (*L*. *interrogans*, serogroup Canicola), CG6 (*L*. *interrogans*, serogroup Icterohaemorrhagiae) and CG73 (*L*. *kirschneri*, serogroup Pomona) (Fig 1, S1 Table).

**Fig 2:**
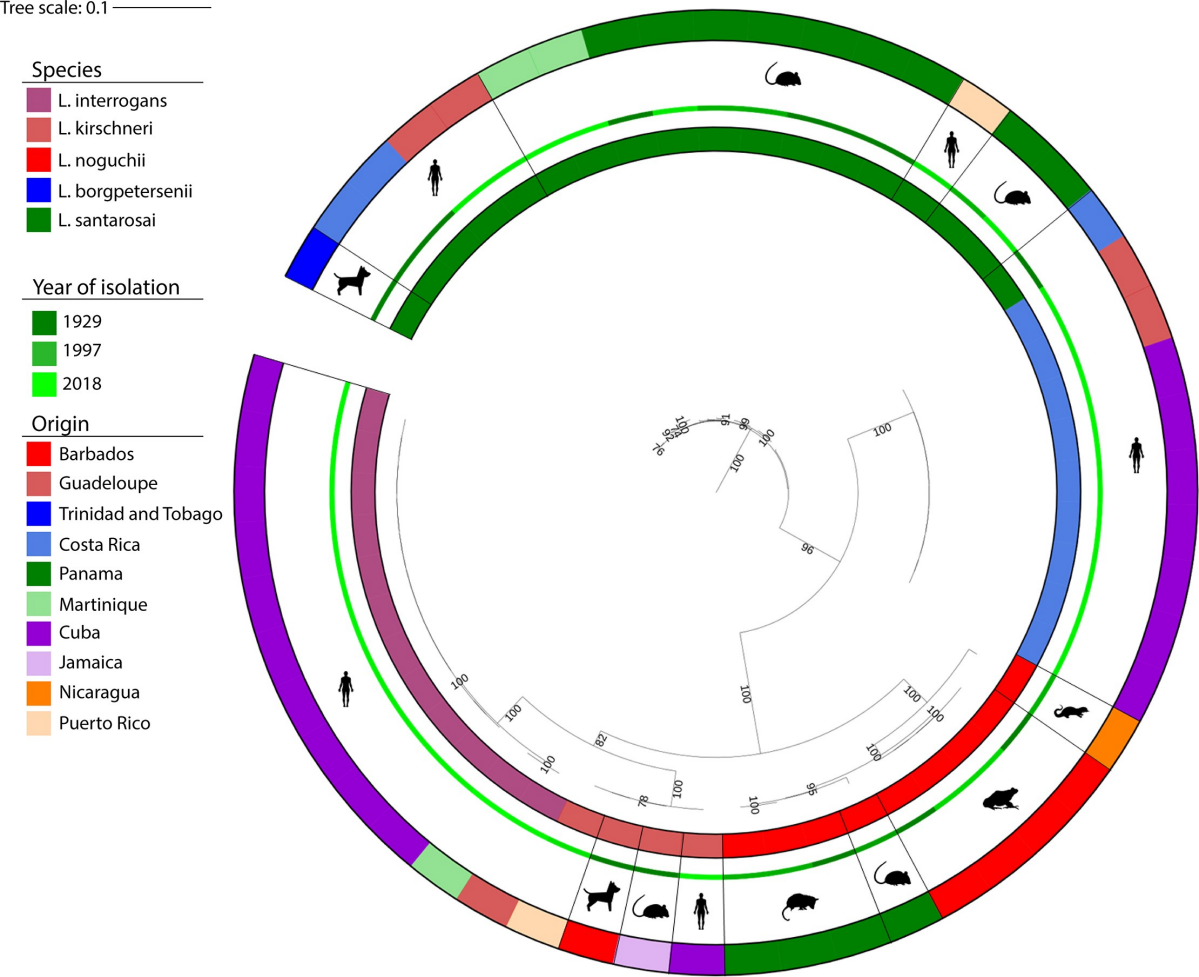
Phylogeny of all available *Leptospira* strains isolated from Caribbean islands and countries in Central America (n = 53). Maximum likelihood bootstrapping was used to generate the phylogenetic tree based concatenated house-keeping genes, that are part of MLST 1 scheme (3,111 bp) [10]. The sequences were extracted from the publically available database hosted at pubMLST and Institut Pasteur MLST (S3 Table). The color codes from the inner to other circles represent species, isolation year, and origin. The samples were isolated from different hosts, as indicated by the silhouettes (human, dog, amphibian, rat/rodent, opossum, and weasel).

**Fig 3:**
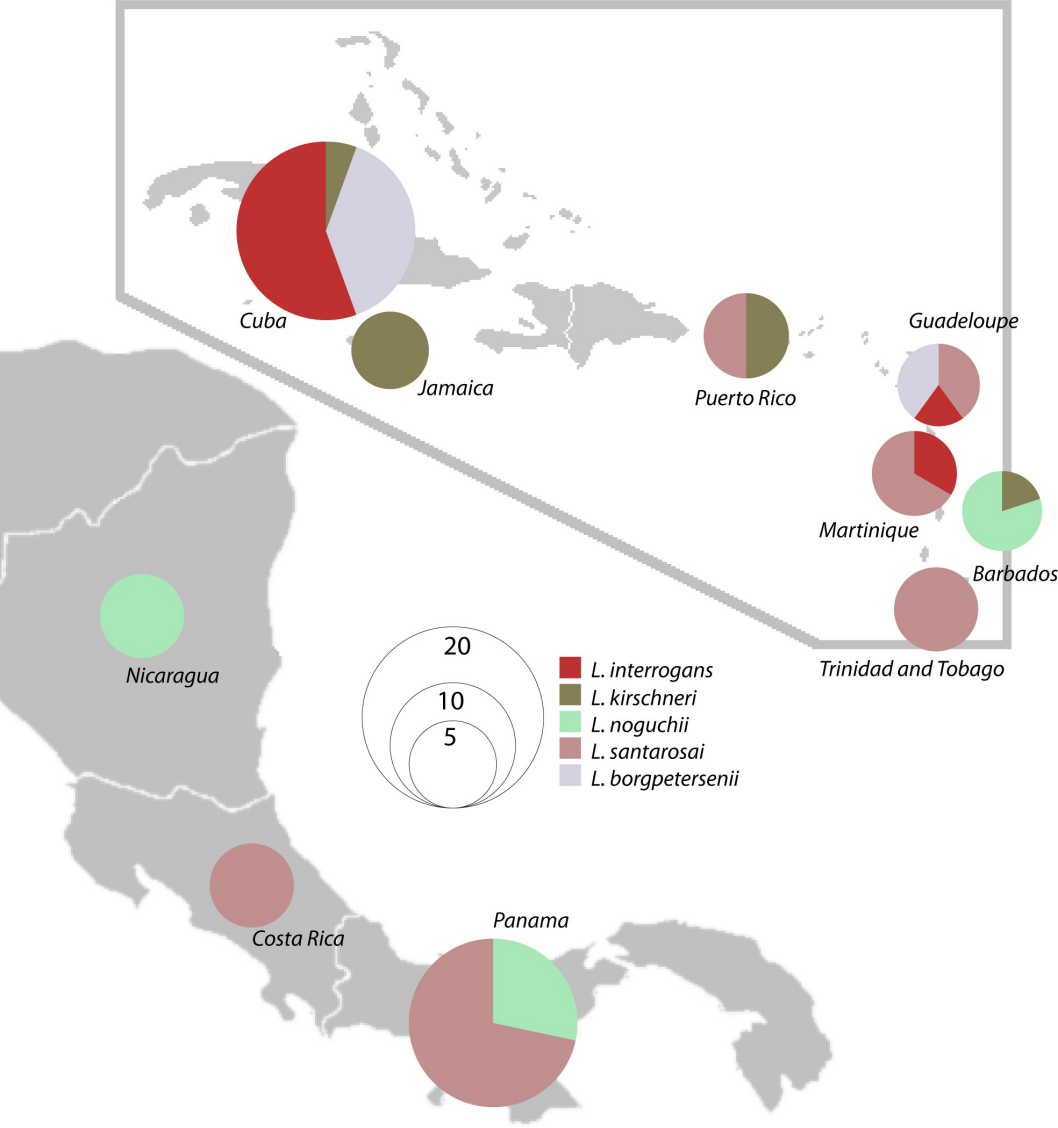
Distribution of *Leptospira* species in Caribbean islands and Central America.

Since there is a limited amount of core genome sequences in this particular geographic region, we have translated the whole genome data of the examined clinical samples into MLST 1, which allowed us to compare the genetic data obtained in Cuba with other Caribbean islands and countries in Central America (n = 53; Fig 2; S3 Table). Even though the sample size is limited, there is an evident difference in the species distribution in Cuba and in neighboring countries (Fig 3). While the Caribbean islands and Central America are characterized as having a high frequency of *L*. *santarosai* [11] we did not identify this species in Cuba. *L*. *santarosai* was detected in five countries as the most predominant species (out of 10; Costa Rica, Trinidad and Tobago, Guadeloupe, Martinique and Panama) isolated from dogs, humans and rodents. On the other hand, *L*. *interrogans*, the most common *Leptospira* species from human infections around the world, was detected only in Cuba (52.6%, 10/19), Guadeloupe (20%, 1/5) and Martinique (25%, 1/4). Cuba, according to these data, is the country with the highest frequency of *L*. *interrogans* in this geographic region.

## Discussion

All clonal groups (cgCGs) that were found in Cuba in the course of this study were described previously among samples typed by cgMLST (n = 922 at the time of writing) and were identified in multiple location across the globe (South America, Europe, Asia, Australia and Africa). However, compared to other Caribbean islands and countries in Central America, Cuban genotypes were unique. For example, the serogroup Canicola has not been isolated in a 10-year period in either Martinique or Guadeloupe [12]. Interestingly, serogroup Icterohaemorrhagiae, which has been identified as the most prevalent serogroup in the majority of countries worldwide, was not detected among the recent Cuban isolates. However, the vax-SPIRAL^®^ vaccine registered in 1998 was generated based on the most prevalent serogroups at that time, including serogroups Canicola, Pomona and Icterohaemorrhagiae. The introduction of this multivalent vaccine may have influenced circulating serogroups in the following years. The only exception was cgCG15 that has been detected in both Guadeloupe and Cuba. Similarly, the distribution of *Leptospira* species in Cuba (i.e. the predominance of *L*. *interrogans* species) was similar to that of the majority of other countries across the globe. However, Caribbean islands and countries in Central America are characterized as having a high frequency of *L*. *santarosai* instead (Fig 3) [6, 11]. In term of infectious diseases, the circulation of the same predominant bacterial strains among neighboring countries or islands can be explained by introduction of rodents or any reservoir hosts by trade, especially via ships. The exchange between Cuba and neighboring islands had been scarce compared with the rest of the countries in the area during the period of strain collection. This may explain why the distribution of the predominant leptospiral species differs significantly from other countries. However, the prevalence of *Leptospira* species found in our study could be biased by the small amount of the examined strains.

Apart from the historical strains, all of the examined Cuban strains were isolated recently from human patients, however, the most prevalent clonal group, cgCG28 (associated with serogroup Canicola), was also isolated from rodents, dogs and pigs in Europe and South America (Institut Pasteur MLST). The second predominant clonal group, cgCG15 (associated with serogroup Ballum), was isolated from rodents from across the globe. Finally, cgCG73 and cgCG5 (both associated with serogroup Pomona) were isolated from rodents, dogs, pigs and cows. As the most predominant CGs in Cuba were CG28 and CG15, we hypothesize that possible source of infection in the country could be dogs, rodents, and pigs. While determination of the ecologic reservoir of leptospires offers important evidence on the source of human infection to help enable control of the disease; additional studies are needed in order to better understand this phenomenon in Cuba and the Americas. Given that cgMLST was recently proposed (2019), the geographical distribution and dissemination pattern of the cgCGs is not yet fully explored, however the wide use of this highly discriminatory method should provide insights into the molecular epidemiology of leptospirosis in the Americas in the future.

When comparing the recently isolated strains with the historical strains used for the vaccine development, there is no significant difference in regard to the phylogeny. Even though we have examined a limited number of samples (n = 19), we have found representatives of the most frequent serogroups described by previous national studies, e.g. Ballum, Pomona, and Canicola, thus confirming that our data set is representative of the strains that had been most frequently circulating in Cuba. Interestingly, since 1996, the prevalence of both Pomona and Canicola has decreasing tendencies, however, the serogroup Ballum has been increasing continuously [13–17]. The bacterins-based vaccine (vax-SPIRAL^®^) introduced in Cuba in 1998 was designed against serogroup Canicola, Pomona and Icterohaemorrhagiae (Fig 1, S1 Table) [4]. Vax-SPIRAL^®^ is recommended to be administrated twice per year for any Cuban people older than 15 years at risk of *Leptospira* exposure. However, this vaccine lacks serogroup Ballum, one of the most frequent serogroups in our examined sample set. This is an important aspect to highlight in the context of National and Regional Leptospirosis Control Programs given the plausible appearance of vaccine insufficiency.

We recognize two main limitations in the present report. The low number of *Leptospira* strains sequenced from Cuba and Americas does not allow for deeper understanding of molecular epidemiology of leptospirosis in the region. Moreover, the inability to obtain clinical and epidemiological information from infected patients precludes analysis about correlations between *Leptospira* genetic patterns and patient characteristics. However, this report shows, for the first time, the molecular characterization of pathogenic *Leptospira* in Cuba and provides insight into the epidemiology and control programs of leptospirosis. These genomic data can also be helpful in developing new diagnostic tools for better detection of the *Leptospira* strains circulating in Cuba.

## Supporting information

S1 Table *Leptospira* strains collected from patients in Cuba during 2008–2012 and historical strains of *Leptospira* used for the original vaccine development. (DOCX)

S2 Table NGS statistics and number of core genes tagged. (DOCX)

S3 Table All *Leptospira* strains isolated from Caribbean islands or countries in Central America with available sequencing data. (DOCX)
