# Supplementary material for: A first insight into the genomic diversity of Leptospira strains isolated from patients in Cuba
Source: PLoS One. 2020 Feb 27;15(2):e0229673. doi: 10.1371/journal.pone.0229673 (PMC7046204; doi:10.1371/journal.pone.0229673)
Supplement: S1 Table — (DOCX) [file pone.0229673.s001.docx]

**S1 Table. *Leptospira* strains collected from patients in Cuba during 2008-2012 and historical strains of *Leptospira* used for the original vaccine development.**

| **BIGSdb id** | **Species** | **Serogroup** | **Serovar** | **Isolation year** | **Province** | **Host** | **Sequencing types (STs)** | **Clonal group (cgCG)** ^a^ |
| --- | --- | --- | --- | --- | --- | --- | --- | --- |
| **817** | *L. borgpetersenii* | Ballum | Ballum | 2012 | Holguin | Human | 731 | 15 |
| **936** | *L. borgpetersenii* | Ballum | Arborea | 2011 | Holguin | Human | 715 | 15 |
| **937** | *L. borgpetersenii* | Ballum | Arborea | 2009 | Holguin | Human | 716 | 15 |
| **938** | *L. interrogans* | Canicola | Canicola | 2010 | La Habana | Human | 717 | 28 |
| **939** | *L. interrogans* | Canicola | Canicola | 2010 | Holguin | Human | 242, 718 ^b^ | 28 |
| **940** | *L. interrogans* | Canicola | Canicola | 2009 | Holguin | Human | 242, 718 ^b^ | 28 |
| **941** | *L. interrogans* | Canicola | Canicola | 2009 | Holguin | Human | 242, 718 ^b^ | 28 |
| **942** | *L. interrogans* | Canicola | Canicola | 2011 | Holguin | Human | 718 | 28 |
| **943** | *L. interrogans* | Canicola | Canicola | 2011 | Holguin | Human | 242, 718 ^b^ | 28 |
| **958** | *L. interrogans* | Pomona | Pomona | 2012 | Holguin | Human | 731 | 5 |
| **959** | *L. interrogans* | Canicola | Canicola | 2011 | Holguin | Human | 242, 718 ^b^ | 28 |
| **960** | *L. kirschneri* | Pomona | Mozdok | 2011 | Holguin | Human | 733 | 73 |
| **961** | *L. interrogans* | Canicola | Canicola | 2011 | Holguin | Human | 734 | 28 |
| **962** | *L. borgpetersenii* | Ballum | Kenya | 2010 | Holguin | Human | 578 | 15 |
| **963** | *L. borgpetersenii* | Ballum | Arborea | 2009 | Holguin | Human | 735 | 15 |
| **964** | *L. borgpetersenii* | Ballum | Arborea | 2010 | Las Tunas | Human | 736 | 15 |
| **965** | *L. borgpetersenii* | Ballum | Sarmini | 2008 | Las Tunas | Human | 737 | 15 |
| **966** | *L. interrogans* | Canicola | Canicola | 2011 | Holguin | Human | 738 | 28 |
| **967** | *L. kirschneri* | Pomona | Mozdok | 2011 | Holguin | Human | 739 | 73 |
| **791**^c^ | *L. kirschneri* | Pomona | Mozdok | 1990 | Villa Clara | Rat | 763 | 73 |
| **792** ^c^ | *L. interrogans* | Canicola | Canicola | 1988 | La Habana | Calf | 764 | 28 |
| **793** ^c^ | *L. interrogans* | Icterohaemorrhagiae | Copenhagenni | 1991 | La Habana | Rat | 765 | 6 |

^a^ cgCGs are defined by a single-linkage clustering threshold of 40 allelic mismatches [6].

^b^ Multiple STs assignments were caused by the absence of sequencing data for several loci in the given samples that distinguish between the given STs.

^c^ Historical strains used for the original vaccine development.
