# Supplementary material for: A first insight into the genomic diversity of Leptospira strains isolated from patients in Cuba
Source: PLoS One. 2020 Feb 27;15(2):e0229673. doi: 10.1371/journal.pone.0229673 (PMC7046204; doi:10.1371/journal.pone.0229673)
Supplement: S2 Table — (DOCX) [file pone.0229673.s002.docx]

**S2 Table. NGS statistics and number of core genes tagged.**

| **BIGSdb id** | **No. of contigs** | **Mean length of contigs (nt)** | **N50 length of contigs (nt)** ^a^ | **Loci tagged** |
| --- | --- | --- | --- | --- |
| **817** | 119 | 32 811 | 62 425 | 553 |
| **936** | 311 | 12 008 | 21 158 | 553 |
| **937** | 243 | 16 038 | 34 422 | 553 |
| **938** | 359 | 12 632 | 24 553 | 552 |
| **939** | 396 | 11 249 | 18 243 | 553 |
| **940** | 290 | 16 040 | 38 893 | 553 |
| **941** | 256 | 18 138 | 41 464 | 553 |
| **942** | 257 | 18 045 | 38 471 | 553 |
| **943** | 284 | 16 346 | 39 020 | 553 |
| **958** | 258 | 17 442 | 32 048 | 553 |
| **959** | 257 | 18 024 | 35 748 | 544 |
| **960** | 416 | 9 185 | 14 746 | 547 |
| **961** | 462 | 8 671 | 13 522 | 546 |
| **962** | 104 | 37 554 | 67 113 | 553 |
| **963** | 247 | 15 758 | 34 599 | 553 |
| **964** | 154 | 24 754 | 49 201 | 553 |
| **965** | 129 | 29 752 | 61 353 | 553 |
| **966** | 418 | 10 761 | 19 976 | 552 |
| **967** | 371 | 11 127 | 19 150 | 550 |
| **791**^b^ | 227 | 19 074 | 31 041 | 544 |
| **792** ^b^ | 315 | 13 361 | 26 475 | 545 |
| **793** ^b^ | 155 | 29 463 | 53 253 | 545 |

^a^ Minimum N50 of 10,000 nt are required.

^b^ Historical strains used for the original vaccine development.
