# Supplementary material for: A first insight into the genomic diversity of Leptospira strains isolated from patients in Cuba
Source: PLoS One. 2020 Feb 27;15(2):e0229673. doi: 10.1371/journal.pone.0229673 (PMC7046204; doi:10.1371/journal.pone.0229673)
Supplement: S3 Table — (DOCX) [file pone.0229673.s003.docx]

**S3 Table. All Leptospira strains isolated from Caribbean islands or countries in Central America with available sequencing data.**

| **BIGSdb id** | **Species** | **Serogroup** | **Serovar** | **Isolation year** | **Origin** | **Host** | **Source** |
| --- | --- | --- | --- | --- | --- | --- | --- |
| **84** | *L. kirschneri* | Autumnalis | Bim | NA | Barbados | Dog | Institute Pasteur MLST |
| **85** | *L. borgpetersenii* | Ballum | Castellonis | 2008 | Guadeloupe | Human | Institute Pasteur MLST |
| **86** | *L. borgpetersenii* | Ballum | Castellonis | 2008 | Guadeloupe | Human | Institute Pasteur MLST |
| **87** | *L. interrogans* | Icterohaemorrhagiae | Icterohaemorrhagiae | 2011 | Guadeloupe | Human | Institute Pasteur MLST |
| **88** | *L. santarosai* | Unknown | Unknown | 2007 | Guadeloupe | Human | Institute Pasteur MLST |
| **89** | *L. santarosai* | Unknown | Unknown | 2004 | Guadeloupe | Human | Institute Pasteur MLST |
| **90** | *L. santarosai* | Unknown | Unknown | NA | Trinidad and Tobago | Dog | Institute Pasteur MLST |
| **91** | *L. santarosai* | Javanica | Arenal | NA | Costa Rica | Human | Institute Pasteur MLST |
| **92** | *L. santarosai* | Javanica | Arenal | NA | Costa Rica | Human | Institute Pasteur MLST |
| **93** | *L. santarosai* | Javanica | Arenal | NA | Costa Rica | Human | Institute Pasteur MLST |
| **96** | *L. santarosai* | Shermani | Shermani | NA | Panama | Rat | Institute Pasteur MLST |
| **429** | *L. noguchii* | Australis | Bajan | 1991 | Barbados | Amphibian | Institute Pasteur MLST |
| **445** | *L. santarosai* | Grippotyphosa | Canalzonae | 1966 | Panama | Rodent | Institute Pasteur MLST |
| **449** | *L. noguchii* | Australis | Barbudensis | NA | Barbados | Unknown | Institute Pasteur MLST |
| **487** | *L. noguchii* | Australis | Bajan | 1991 | Barbados | Amphibian | Institute Pasteur MLST |
| **496** | *L. santarosai* | Grippotyphosa | Canalzonae | 1966 | Panama | Rodent | Institute Pasteur MLST |
| **510** | *L. noguchii* | Australis | Barbudensis | NA | Barbados | Amphibian | Institute Pasteur MLST |
| **515** | *L. noguchii* | Australis | Unknown | NA | Panama | Rodent | Institute Pasteur MLST |
| **526** | *L. santarosai* | Celledoni | Unknown | 2011 | Martinique | Human | Institute Pasteur MLST |
| **527** | *L. santarosai* | Celledoni | Unknown | 2011 | Martinique | Human | Institute Pasteur MLST |
| **817** | *L. borgpetersenii* | Ballum | Ballum | 2012 | Cuba | Human | Institute Pasteur MLST |
| **824** | *L. interrogans* | Icterohaemorrhagiae | Unknown | 2018 | Martinique | Human | Institute Pasteur MLST |
| **936** | *L. borgpetersenii* | Ballum | Arborea | 2011 | Cuba | Human | Institute Pasteur MLST |
| **937** | *L. borgpetersenii* | Ballum | Arborea | 2009 | Cuba | Human | Institute Pasteur MLST |
| **938** | *L. interrogans* | Canicola | Canicola | 2010 | Cuba | Human | Institute Pasteur MLST |
| **939** | *L. interrogans* | Canicola | Canicola | 2010 | Cuba | Human | Institute Pasteur MLST |
| **940** | *L. interrogans* | Canicola | Canicola | 2009 | Cuba | Human | Institute Pasteur MLST |
| **941** | *L. interrogans* | Canicola | Canicola | 2009 | Cuba | Human | Institute Pasteur MLST |
| **942** | *L. interrogans* | Canicola | Canicola | 2011 | Cuba | Human | Institute Pasteur MLST |
| **943** | *L. interrogans* | Canicola | Canicola | 2011 | Cuba | Human | Institute Pasteur MLST |
| **958** | *L. interrogans* | Pomona | Pomona | 2012 | Cuba | Human | Institute Pasteur MLST |
| **959** | *L. interrogans* | Canicola | Canicola | 2011 | Cuba | Human | Institute Pasteur MLST |
| **961** | *L. interrogans* | Canicola | Canicola | 2011 | Cuba | Human | Institute Pasteur MLST |
| **962** | *L. borgpetersenii* | Ballum | Kenya | 2010 | Cuba | Human | Institute Pasteur MLST |
| **963** | *L. borgpetersenii* | Ballum | Arborea | 2009 | Cuba | Human | Institute Pasteur MLST |
| **964** | *L. borgpetersenii* | Ballum | Arborea | 2010 | Cuba | Human | Institute Pasteur MLST |
| **965** | *L. borgpetersenii* | Icterohaemorrhagiae | Sarmini | 2008 | Cuba | Human | Institute Pasteur MLST |
| **966** | *L. interrogans* | Canicola | Canicola | 2011 | Cuba | Human | Institute Pasteur MLST |
| **967** | *L. kirschneri* | Pomona | Mozdok | 2011 | Cuba | Human | Institute Pasteur MLST |
| **98** | *L. kirschneri* | Icterohaemorrhagiae | Bogvere | NA | Jamaica | Rat | pubMLST |
| **437** | *L. noguchii* | Australis | Nicaragua | 1965 | Nicaragua | Weasel | pubMLST |
| **102** | *L. noguchii* | Panama | Panama | 1962 | Panama | Opossum | pubMLST |
| **106** | *L. santarosai* | Bataviae | Balboa | 1966 | Panama | Rat | pubMLST |
| **110** | *L. santarosai* | Bataviae | Kobbe | 1962 | Panama | Rat | pubMLST |
| **438** | *L. noguchii* | Pomona | Proechimys | 1952 | Panama | Rat | pubMLST |
| **442** | *L. santarosai* | Mini | Beye | 2003 | Panama | Rat | pubMLST |
| **444** | *L. santarosai* | Grippotyphosa | Canalzonae | 2006 | Panama | Rat | pubMLST |
| **469** | *L. santarosai* | Pomona | Tropica | 1929 | Panama | Rat | pubMLST |
| **576** | *L. santarosai* | Shermani | Shermani | NA | Panama | Rat | pubMLST |
| **717** | *L. noguchii* | Panama | Panama | 1966 | Panama | Opossum | pubMLST |
| **728** | *L. santarosai* | Shermani | Shermani | NA | Panama | Rat | pubMLST |
| **445** | *L. santarosai* | Hebdomadis | Borincana | 2000 | Puerto Rico | Human | pubMLST |
| **465** | *L. kirschneri* | Bataviae | Djatzi | 2000 | Puerto Rico | Human | pubMLST |

NA – not available.
